# Caring Behaviors Inventory-24: translation, cross-cultural adaptation, and psychometric testing for using in nurses and patients

**DOI:** 10.1186/s12912-023-01248-2

**Published:** 2023-03-24

**Authors:** Neda Azimi Khaletabad, Moloud Radfar, Mojgan Khademi, Hamidreza Khalkhali

**Affiliations:** 1 Department of Psychiatric Nursing, School of Nursing and Midwifery, Urmia University of Medical Sciences, Urmia, Iran; 2 Patient Safety Research Center, Clinical Rsearch Institue, School of Nursing and Midwifery, Urmia University of Medical Sciences, Pardis Nazlou. 11 Km of Nazlou Road, Urmia, Iran; 3 Social Determinants of Health Research Center, Lorestan University of Medical Sciences, Khorramabad, Iran; 4 Department of Biostatistics and Epidemiology, School of Medicine, Urmia University of Medical Sciences, Urmia, Iran

**Keywords:** Caring, Behaviors, Psychometrics, Survey and Questionnaire, Nurses, Patients

## Abstract

**Background:**

To measure caring behaviors, it is necessary to have an instrument adapted based on the contextual culture. This study aimed to translate Caring Behaviors Inventory-24 (CBI-24) into Persian and determine its psychometric properties.

**Methods:**

This is a methodological study conducted to translate and then psychometrically test The CBI-24. The forward–backward translation was conducted using the World Health Organization (WHO) model and Wild et al. (2005) approach. The face, content, and construct validity of the inventory were assessed using cognitive interviews (10 nurses and 10 patients), expert panel deliberations (10 experts), and the exploratory and confirmatory factor analyses (300 nurses and 300 patients), respectively. The reliability was determined using the internal consistency (300 nurses and 300 patients) and test–retest method (30 nurses and 30 patients).

**Results:**

After translating the CBI-24 and combining its items, the forward translation was initially conducted and the final backward translation was then sent to the developer for confirmation. The final version of the inventory was prepared after the completion of cognitive interviews. The content validity index of all items was reported to be more than 0.8 and good. The Cohen's kappa coefficient of all items was also shown to be higher than 0.74 and excellent. The factor loading of all items except item 19 was above 0.3. Item 19 was removed since it caused the alpha value of the respectfulness dimension to be 0.32. The Cronbach's alpha and the correlation coefficient of the whole inventory were calculated to be 0.95 and 0.88, respectively.

**Conclusion:**

The Persian version of CBI-24 can be a suitable tool for measuring caring behaviors among patients and nurses.

## Background

Care is an integral part of the nursing profession [1] and is also considered a universal need, phenomenon, and basic concept that affects human relationships [2]. In the theory of human care, Watson (2008) states that care is a moral ideal and unique use of oneself to achieve unity between two people, under the shadow of which healing, inner strength, and self-control flourish [3, 4].

Caring behaviors are not equally understood in different societies [5]. Culture and values affect the understanding of the caring concept [6]. Meeting patients' emotional or psychosocial needs is considered as an important part of care in developed countries, while the main part of care is concentrated on its physical and technical aspects in Iran [7]. The concept of care in nursing practice is non-objective, abstract, and intangible so that there are still many debates over nursing care and its evaluation. Some experts pay attention to the mental and abstract aspects of care, while others evaluate it in accordance with physiological indicators. Therefore, care evaluation is essential because it is necessary to advance the care knowledge and its results can be observed and evaluated in patients' health status and performance [2]. In addition, patients and nurses have different perceptions of medical care. Nurses consider caring behaviors related to respect, privacy and dignity of the patient important, but patients focus on behaviors such as professional knowledge and skills to perform nursing activities and safety [8]. Differences between the patients' and the nurses' perception of caring behaviors can cause patient dissatisfaction. Therefore, the evaluation and comparison of these behaviors according to patients and nurses' perspectives can provide better feedback for health workers, especially nursing managers [9]. The identification of instruments for measuring care and analyzing the impact of caring behaviors on nurses and patients can direct nursing performance and ensure that nursing care is the main priority in health systems [10].

The effective measurement of care process is also of great importance in nursing research [5]. Researchers have used different instruments to study caring behaviors [11], all of which have been developed based on researchers' interests and thoughts and sometimes based on care theories. Accordingly, these instruments have ultimately caused the development and evolution of care science [12]. In most of the available instruments used for measuring caring behaviors, target groups are composed of students [5, 13], patients, and nurses [7, 11, 14]. Based on a literature review conducted in Iranian databases, there is a strong desire for using non-Persian instruments for assessing caring behaviors, which can be due to the lack of appropriate Persian instruments in this regard. Concerning the development of nursing science and the need to use evidence-based sources, the translation and psychometric assessment of existing instruments are inevitable [10]. Some of these instruments have already been translated into Persian and psychometrically assessed in the Iranian context [9, 12, 15–21]. Most related studies have not stated all the steps of psychometric assessment. Some researchers have limited themselves to only measuring the content validity of the instrument [9, 15, 16] and some have only measured the reliability [12, 17]. Therefore, there is an essential need for a valid Persian tool, which can be utilized in studies focused on the evaluation of caring behaviors.

The globalization and migration have increased the population diversity around the world. This increase in population diversity and the need for conducting cross-cultural and multinational research indicate a strong need for health care researchers to have access to reliable and valid instruments validated in populations with various cultures or languages. The findings of cross-cultural studies may have major clinical implications for physicians, nurses, and other healthcare professionals as the delivery of quality care depends on an accurate assessment and deeper understanding of an individual's cultural, linguistic, and ethnic background [22]. In addition, psychometrics and cross-cultural adaptation of an instrument contribute to the validity of the original version of that instrument.

The Caring Behaviors Inventory (CBI) is a 42-item tool mainly developed by Wolf et al. (1998) and modified after development. The Caring Behaviors Inventory-24 (CBI-24) is the short version of the 42-item CBI and has been designed by Wu et al. (2006). The CBI-24 is a new tool used to assess patients and nurses' perceptions of caring behaviors [23]. The advantages of this tool include its novelty, standard number of items, and usability for both patients to get their opinions about caring behavior and for nurses to examine caring behaviors based on their perspectives. The 42- and 16-item CBIs have been both translated and psychometrically tested in Iran [19]. Nonetheless, the 24-item CBI, which covers all of the caring behavior dimensions and is more balanced and shorter than the other two versions, has not been translated and psychometrically assessed. It has been shown that the length of the items of an instrument is an important issue when using it. For instance, questionnaires with longer questions are not completely answered most of the time and it seems that their final questions are answered with less accuracy [11]. Johansen et al. (2013) reported that people with a low level of education complete long questionnaires to a lesser extent [24]. Therefore, the present study aimed to translate the CBI-24 into Persian and then assess its psychometric properties in an Iranian context.

## Methods

This is a methodological study conducted from May 2019 to July 2020. The population of study consists of nurses, head nurses, and patients hospitalized at four teaching hospitals, Imam Khomeini with 638 beds and 725 nurses, Motahari with 298 beds and 320 nurses, Taleghani with 219 beds and 302 nurses, Seyyed al-Shohda with 145 and 234 nurses. Samples were selected from the patients hospitalized in hematology, dialysis, surgery, internal medicine, nephrology, cardiology, gynecology, pulmonology, gastroenterology, and intensive care departments using convenience sampling. The inclusion criteria for nurses consisted of the followings: (a) having at least a bachelor's degree in nursing and (b) having at least six months of work experience in one of the above departments. Inclusion criteria for patients included the followings: (a) being over 18 years of age, (b) being hospitalized during the study period, (c) having stable physiological health status (d) having no cognitive or psychological disorders, and (e) having the ability to communicate in Persian or Turkish. In contrast, exclusion criteria for patients consisted of the occurrence of significant changes in physiological or psychological health status during the completion of the questionnaire. The exclusion criteria for both patients and nurses included the followings: (a) unwillingness to participate in the study and (b) incomplete completion of the questionnaire.

The CBI-24 is scored based on a 6-point Likert scale from "Never = 1" to "Always = 6". This inventory measures caring behaviors in four dimensions of assurance, knowledge and skill, respectfulness, and connectedness [23]. This study was conducted in two phases of translation and psychometric assessment.

### Phase 1: translation

After obtaining approval from the main developer of the inventory (Zane Robinson Wolf), the translation was conducted in two stages using the World Health Organization (WHO) model [25] and the Wild et al. (2005) approach [26]. At the first stage, the forward translation (English to Persian) was performed by three translators; an Iranian professor of English Language and Linguistics and two Iranian nursing faculty members who were familiar with the concept of caring behaviors and English language as well. Then the transcripts were combined by the first author under the supervision of the second and the third authors, and the combined version was reviewed, compiled, and approved in three sessions by a panel of experts.

The final transcript of the forward translation was translated into English by two independent English translators who were familiar with Iranian culture. Then the final transcript of the backward translation was sent to the developer and his recommendations were adopted. After applying developer's recommendations, the final revised transcript of the translation was sent to him again. After obtaining his approval, minor corrections were made in the Persian version of the transcript.

The first author conducted cognitive face-to-face interviews with 10 nurses and 10 patients from different socioeconomic statuses and age groups. In order to prevent participants from being exhausted, the interviews were conducted several times in different work shifts. The items were read for them in a correct order and they were then asked to firstly say what they thought about the question and what came to their mind when they heard a particular word, and secondly explain how they chose their answers. After applying the comments of the target groups, the final Persian version of the CBI-24 was prepared for psychometric testing. Each copy of the inventory was separately encoded using a serial number and the necessary documentation was conducted about the translation.

### Phase 2: psychometric assessment

The face validity of the inventory was assessed by having interviews with 10 nurses and 10 patients. The Content Validity Index (CVI) was evaluated by sending the inventory to 10 experts, who were familiar with psychometric testing. The experts were asked to determine the relevance of items based on a 4-point Likert scale (Not relevant = 1, Relatively relevant = 2, Almost relevant = 3, Completely relevant = 4) [27]. Items with a score of greater than 0.79 were considered appropriate; those with a score of 0.70- 0.79 required to be modified; and those with a score of less than 0.70 were removed.

$$CVI=\frac{Number\;of\;experts\;who\;gave\;the\;scores\;of\;3\;or\;4\;to\;the\;item}{Total\;number\;of\;experts}$$

The modified Cohen's kappa, which indicates the degree of agreement between the experts, was also calculated using the following formula [28]:

$$k=\frac{I\_CVI-Pc}{1-Pc}$$

*I-CVI* = *The CVI of the item*

*Pc = The probability of chance agreement*

$$Pc=[N!/A!(N-A)!]\times 0.5N$$

In the above formula, *N* is the number of experts and *A* is the number of experts agreed that the item is relevant (those who gave the scores of 3 or 4). Modified Cohen's kappa values of higher than 0.74 were considered excellent; those between 0.6 and 0.74 were considered good; and those less than 0.6 were considered fair.

In this study, Exploratory Factor Analysis (EFA) and Confirmatory Factor Analysis (CFA) were utilized to assess the construct validity. To conduct the CFA, the minimum sample size was considered at least 300 people, which is 6–10 times more than the model parameters [29]. Accordingly, the inventory was given to 300 nurses and 300 patients. The EFA was also conducted using two tests of Kaiser − Meyer − Olkin (KMO) and Bartlett sphericity, based on which the adequacy of sample size and appropriateness of correlation coefficients between the items were determined and approved. The results of CFA are separately presented for the study variables using the LISREL software (Scientific Software International Inc., Chicago, IL, USA). It should be also noted that the factor loading should be greater than 0.3 for reducing the variables and considering them as latent variables. In the examination of every single model, the main question is whether these measurement models are appropriate. To answer this question, the chi-squared good-of-fit test and other criteria of model fit assessment should be checked. Therefore, the Goodness of Fit Indices (GFIs) with the following results shows a good-fitting model; lower χ2/df good-of-fit test because this test gives a difference between data and model; the Root Mean Square Error of Approximation (RMSEA) should be low because this is the Mean Squared Error (MSE) of the model; the Minimum Value of Chi-Square/Degree of Freedom Ratio (CMIN/DF) should be less than 3.0; the RMSEA should be less than 0.08, and other indices should be closer to one [30].

The reliability was assessed using the methods of test–retest (consistency across time) and internal consistency (consistency across items). To examine internal consistency, Cronbach's alpha coefficient was calculated. For this purpose, the inventory was completed in pairs by 300 nurses and 300 patients. To examine test–retest reliability, the inventory was filled in twice by 30 nurses and 30 patients. In the retest phase of the test–retest reliability assessment, the very same nurses and patients re-completed the inventory a week and 48 h later, respectively.

Data were collected in different shifts by the first author. First, the inventory was given to the patient and he/she was requested to answer the questions based on the performance of his/her nurse. For illiterate patients, the items were read with no change in the meaning and their actual opinions were recorded. The inventory was then given to the very same nurse who was caring for the client and this nurse was asked to answer the questions.

Before completing the questionnaires after making effective communication, the researcher explained the study objectives to the participants. It was emphasized that participation in the study was completely voluntary and if they are not eager to continue participation in the study, there would be no disruption in their treatment process. Participants were given enough time to complete the questionnaires. The researcher stays with them to return the completed questionnaires if they wish. All participants returned the completed questionnaires and there were no missing data.

Data were managed and analyzed using the SPSS Statistics for Windows, version 16.0 (SPSS Inc., Chicago, Ill., USA) and the LISREL software (Scientific Software International Inc., Chicago, IL, USA).

## Results

### Translations, cultural adaptation, and expert panel review

The translation process was performed systematically. The semantic, conceptual, and idiomatic equivalence of phrases was discussed during the translation. At the forward translation, most of the questions were straightforward so that the translations were easily combined. However, there were differences between the translators' translations in items 4, 6, 12, 20, and 22. Accordingly, these items were examined by a panel of experts. After holding three sessions, the panel came up with a single translation for the above items.

At the backward translation, one of the translators did not correctly translate item 16 (*Visiting the patients voluntarily*). The examination of the original English version of the inventory clearly indicated that this was a translation error. Consequently, the relevant translator was informed of the translation error and then asked to review and correct it (*Looking the patients up voluntarily*). The backward translation of the inventory was sent to the developer. Based on the developer's perspective, item 16 did not conform to the original version of the inventory and items 14, 19, 20, and 23 required minor corrections but were in line with the original version. The expert panel held a session again, during which the necessary changes were made to fully correct the above items and these corrections led to a simplification and better understanding of the items. The forward and backward translation of item 16 was conducted again (Table 1). Then the inventory was re-sent to the developer with final corrections.

**Table 1:** Items modified in backward translation according to Wolf approach

Item No	Backward translation items sent to Wolf	Item modified according to Wolf
14	Letting patients participate in self-care program	Letting patient participate in his/her care plan
16	Looking the patients up voluntarily	Checking on patients voluntarily
19	Providing the stated and none-stated needs of the patient	Providing the stated and unstated needs of the patient
20	A quick response to the patient's call alert	Quickly responding to the patient's call
23	On time treatment and medications	Being On time treatment and medications

The changes mentioned by the developer for items 14, 19, 20 and 23 were made after the expert panel review, which led to simplification and better understanding of the items. Forward and backward translation of item 16 was done again.

In the cognitive interviews, participants stated that most of the items were relevant and understandable. However, in-depth interviews were needed for items 3, 5, 7, 11, and 24. In the second cognitive interview, a single translation was reached for the above items and the Persian version of the inventory was finally prepared for psychometric testing.

### Psychometric analysis

A total of 300 patients and 300 nurses participated in this study. The mean age of nurses and patients was 30.89 ± 5.93 and 42.20 ± 16.80, respectively. The qualitative-cognitive characteristics of the samples are presented in Table 2.

**Table 2:** Qualitative-cognitive characteristics of nurses and patients

**Variable**		**Nurses** **No. (%)**	**Variable**		**Patients** **No. (%)**
**Gender**	Male	84 (28)	**Gender**	Male	156 (52)
	Female	216 (72)		Female	144 (48)
**Marital status**	Married	117 (59)	**Marital status**	Married	213 (71)
	Single	113 (37.7)		Single	74 (24.7)
	Other	10 (3.3)		Other	13 (4.3)
**Type of employment**	Official	49 (16.3)	**Occupational status**	Employed	109 (36.3)
	contract	26 (8.7)		Retired	47 (15.7)
	contractual	99 (33)		housewife	104 (34.7)
	Other	126 (42)		Unemployed	40 (13.3)
**Education**	Bachelor d	281 (93.7)	**Education**	Literacy	97 (32.3)
				Diploma	129 (43)
	Master d	19 (6.3)		Bachelor d	65 (21.7)
				Master d	9 (3)
**Work shift**	Fixed morning shift	33 (11)	**Hospitalization ward**	Pulmonary	32 (10.7)
				surgery	67 (22.3)
				Hemodialysis	8 (2.7)
	Morning or evening shift	20 (6.7)		oncology	25 (8.3)
				gastrointestinal	25 (8.3)
				Nephrology	24 (8)
	rotating shift	247 (82.3)		infectious	30 (10)
				poisoning	11 (3.7)
				Cardiac	58 (1903)
				Gynecology	20 (6.7)

According to the data provided in the above table, most of the nurses were female and married. Moreover, the majority of them had a bachelor's degree and a rotating shift. The majority of patients were male, married, employed, and had a diploma.

### Validity assessment

The face validity of the inventory was assessed using the cognitive interviews by checking criteria, including ease of completion, grammar and spelling, transparency, and the writing style of the items.

The CVIs of all items ranged from 0.8 to 1 and were therefore indicated to be acceptable. The overall CVI of the inventory was calculated to be 0.94, which is at a good level. Furthermore, the Modified kappa coefficient (K^✶^) of fourteen items was reported to be 1 and those of other items were higher than 0.74, which is excellent (Table 3).

**Table 3:** Content validity index and modified Kappa coefficient

Item No	CVI	I-CVI	PC	K*
1	1	1	0.000976563	1
2	1	1	0.000976563	1
3	1	1	0.000976563	1
4	1	1	0.000976563	1
5	1	1	0.000976563	1
6	1	1	0.000976563	1
7	0.9	1	0.009765625	1
8	0.9	0.9	0.009765625	0.8990
9	0.8	0.9	0.043945313	0.8990
10	1	0.8	0.000976563	0.7908
11	1	1	0.000976563	1
12	1	1	0.000976563	1
13	0.9	1	0.009765625	1
14	1	0.9	0.000976563	0.8990
15	1	1	0.000976563	1
16	0.9	1	0.009765625	1
17	0.8	0.9	0.043945313	0.8990
18	0.9	0.8	0.009765625	0.7908
19	0.8	0.9	0.043945313	0.8990
20	0.8	0.8	0.043945313	0.7908
21	1	0.8	0.043945313	0.7908
22	09	1	0.009765625	1
23	1	0.8	0.000976563	0.8990
24	1	1	0.000976563	1

k* is the symbol of modified kappa coefficient

The CVIs of all items ranged from 0.8 to 1 and were therefore indicated to be acceptable. The overall CVI of the inventory was calculated to be 0.94, which is at a good level. Furthermore, the Cohen's kappa coefficient of fourteen items was reported to be 1 and those of other items were higher than 0.74, which is excellent (Table 3).

Two tests of KMO and Bartlett sphericity were utilized to conduct the EFA, based on the results of which the adequacy of sample size and appropriateness of correlation coefficients between the items were determined and approved. Based on the results, the KMO value (a measure of sampling adequacy) is 0.92 and the significance level of the Bartlett sphericity test is less than 0.001. Therefore, the correlation matrix obtained for the sample group in the EFA can be justified (Table 4).

**Table 4:** The results of KMO and Bartlett's sphericity test for determining the validity of the CBI-24

**Kaiser–Meyer–Olkin**	**Bartlett's Test of Sphericity**	**DF**	***P*-Value**
0.92	1.30	276	< 0.001

Based on the results, the KMO value (a measure of sampling adequacy) is 0.92 and the significance level of the Bartlett sphericity test is less than 0.001. Therefore, the correlation matrix obtained for the sample group in the EFA can be justified.

In order to obtain a meaningful structure from factor loadings, the extracted factors were transferred to new axes that are placed at right angles to each other based on conventional methods and using orthogonal rotation. Item19 was removed after several times of factor analysis, extraction of multiple factors, comparison of extracted factors with the theoretical structure of the inventory and the existing theoretical foundations, consideration of the factor analysis assumptions, and examination of the commonalities of items. The main reason for removing item 19 was its factor loading of less than 0.3.

The final factor analysis that was conducted using Varimax rotation led to the extraction of four factors. The main statistical indices in conducting EFA for each factor are separately extracted and show the items related to each factor. According to the data provided in Table 5, the eigenvalue of four factors is greater than 1 and the percentage of common variance coverage between the variables for these four factors together explains 72.66% of the total variance of the variables, which indicates the appropriateness of factor validity of items.

**Table 5:** Factor load of EFA

Dimensions	**ItemNo**	**The first component**	**The second component**	**The third component**	**The fourth component**
Respectfulness	1	0.54	0.32	0.34	0.49
	3	0.70	0.38	0.034	0.10
	5	0.65	0.47	0.11	0.09
	6	0.69	0.33	0.20	0.28
	13	0.73	0.08	0.10	0.38
	19	0.19	0.05	0.16	0.001
	22	0.80	0.25	0.11	0.03
Connectedness	2	0.26	0.68	0.32	0.39
	4	0.44	0.72	0.13	0.012
	7	0.28	0.70	0.23	0.30
	8	0.09	0.73	0.07	0.34
	14	0.14	0.69	0.09	0.46
	17	0.09	0.77	0.36	0.05
knowledge and skill	9	0.03	0.45	0.65	0.30
	10	0.05	0.50	0.68	0.23
	11	0.07	0.35	0.75	0.19
	12	0.01	0.27	0.73	0.11
	15	0.27	0.17	0.59	0.25
Assurance	18	0.05	0.20	0.14	0.74
	20	0.29	0.06	0.09	0.82
	21	0.33	0.05	0.14	0.79
	23	0.41	0.13	0.03	0.74
	24	0.33	0.09	0.11	0.78

According to the data provided in Table 5, the eigenvalue of four factors is greater than 1 and the percentage of common variance coverage between the variables for these four factors together explains 72.66% of the total variance of the variables, which indicates the appropriateness of factor validity of items.

**Fig. 1:**
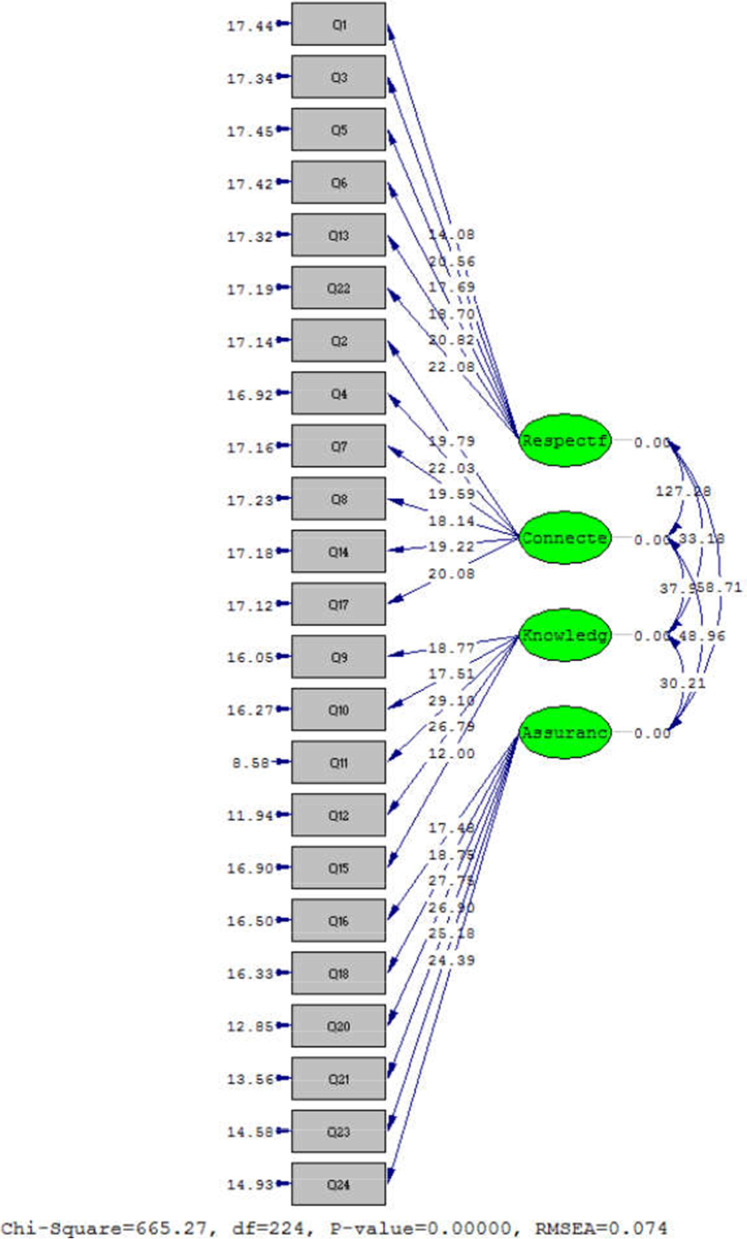
CFA model of the psychometric assessment of the CBI (nurses and patients) in a meaningful state

Some of the most important goodness-of-fit indices in CFA are provided in Table 6, based on which all parameters were shown to be at a very good level and the model had a good fit with the data. This indicates that the items were consistent with the theoretical structure. According to the LISREL software output (Fig. 1), the chi-square value was 665.27. The low value of chi-square indicates a slight difference between the conceptual model and the data obtained. Moreover, the value of RSMEA was 0.074, which is standard and indicates a good model fit. The lower value of RSMEA shows a better model fit. The adaptive fit indices of Comparative Fit Index (CFI), Normed Fit Index (NFI), Relative Fit Index (RFI), and Incremental Fit Index [IFI] showed an excellent model fit. Besides, the absolute indices of the Goodness-of-Fit Index (GFI) and Adjusted Goodness-of-Fit Index (AGFI) are the measures of the relative values of variances and covariances that could be jointly justified by the model. Other indices also showed the model fit to be excellent and standard. The closer the value of GFI and AGFI is to 1, the more appropriate the data fit will be. The values of the above indices were obtained to be 0.92 and 0.91 in this study, which is appropriate. It should be also noted that the values of GFI and AGFI are not affected by the sample size.

**Table 6:** Goodness-of-fit indices in CFA

**Abbreviation**	**The Goodness-of-fit index**	**Acceptable value**	**Observed values**
RMSEA	Root Mean Square Error of Approximation (RMSEA)	< 0.1	0.074
CMIN/DF	Chi-degree freedom	< 3	2.96
IFI	Incremental fit index	> = 0.90	0.92
RFI	Relative fit index	> = 0.90	0.91
NFI	Normed Fit Index	> = 0.90	0.89
GFI	Goodness of fit	> = 0.90	0.92
AGFI	Adjusted Goodness of Fit	> = 0.90	0.88
CFI	Comparative Fit Index	> = 0.90	0.91

The above output shows the significance of the coefficients and parameters of the model in the *t*-value mode, which can be used to confirm or reject the hypotheses. If the amount of *t*-value is greater or less than 1.96, the correlation coefficients in the model will be significant. Therefore, all correlations are significant in the model.

Some of the most important goodness-of-fit indices in CFA are provided in Table 6, based on which all parameters were shown to be at a very good level and the model had a good fit with the data. The value of RSMEA was 0.074, which is standard and indicates a good model fit. The adaptive fit indices of Comparative Fit Index (CFI), Normed Fit Index (NFI), Relative Fit Index (RFI), and Incremental Fit Index all showed an excellent model fit. Besides, the absolute indices of the Goodness-of-Fit Index (GFI) and Adjusted Goodness-of-Fit Index (AGFI) are the measures of the relative values of variances and covariances that could be jointly justified by the model.

### Reliability assessment

Based on the results presented in Table 7, the Cronbach's alpha coefficient of the whole inventory was 0.95. The very same coefficient for the dimensions of knowledge and skill, respectfulness, connectedness, and assurance was calculated to be 0.84, 0.87, 0.89, and 0.90, respectively. The alpha value of the respectfulness dimension was increased to 0.87 by removing item 19. If this item had been kept, the alpha value of the dimension could have been decreased to 0.32. The results of the test–retest reliability assessment showed a high Spearman correlation coefficient, which indicates the reliability of the inventory.

**Table 7:** Reliability of CBI-24 (Cronbach's alpha coefficient and test–retest)

**Dimension**	**Item**	**Cronbach's alpha coefficient**	**Mean (SD)**	**Mean (SD)**	**Spearman correlation coefficient**
			Test	Retest	
Respectfulness	1,3,5,6,13,16, 19 (deleted),22	0.87	43.32 (4.99)	42.12 (4.12)	0.87
Connectedness	2,4,7,8,14,17	0.89	27.01 (3.28)	26.87 (3.10)	0.75
knowledge and skill	12,11,9,15,10	0.84	28.48 (2.56)	29.12 (2.14)	0.89
Assurance	18, 20,21,23,24	0.90	28.24 (2.80)	29.22 (2.54)	0.92
Total	All items except item 19	0.95	127.06 (12.56)	126.52 (11.76)	0.88

Based on the results presented in Table 7, the Cronbach's alpha coefficient of the whole inventory was 0.95. The alpha value of the respectfulness dimension was increased to 0.87 by removing item 19. The results of the test–retest reliability assessment showed a high Spearman correlation coefficient, which indicates the reliability of the inventory.

## Discussion

The present study aimed to translate the CBI-24 into Persian, cross-culturally adapt it, and psychometrically analyze it in an Iranian context. During this study, the Persian version of CBI-24 was prepared for nurses and patients. The results of the psychometric analysis also revealed that the Persian version of CBI-24 is a reliable and valid instrument for measuring caring behaviors among nurses and patients.

### Translation

Translation and psychometric testing of existing instruments in different contexts allows countries and populations to be compared with each other [31]. The aim of translation and psychometric assessment of an instrument is to have it in different languages and therefore use it in different contexts while the same basic concept of its original version is kept unchanged. In this process, the translated version of the instrument must be just as acceptable as its original version. The forward–backward translation is reported by the WHO as an appropriate and standard method of turning the original version of an instrument into versions with different languages [25]. Moreover, several independent translators were recruited at each step based on the recommendations of Wild et al. (2005) to ensure the correct translation of the items throughout the steps of forward–backward translation [26], which led to the enrichment of the translation process.

Patiraki et al. (2014) conducted an international study to understand caring behaviors among nurses and patients from six countries including Finland, Hungary, the Czech Republic, Cyprus, Greece, and Italy. In their study, researchers of each country were asked to translate the questionnaire into their official language with the help of two independent translators based on the MAPI guidelines (MAPI Research Institute, 2009). The equivalence of the CBI-24 was assessed and approved during meetings between researchers from these six countries [32]. In an international study conducted by Palese et al. (2011) in Cyprus, Czech Republic, Finland, Greece, Hungary, and Italy, the CBI-24 and Patient Satisfaction Scale (PSS) were translated into the official language of each country based on the MAPI guidelines [33]. In MAPI linguistic validation method, the instrument is sent to the MAPI Institute after the forward and backward translation so that the institute can compare the original version of the instrument with the final backward English version and then apply the result. Then the instrument can be tested in a real context [34]. The advantage of the WHO model over the MAPI Institute validation method is the use of an expert panel in combining forward and backward translations.

### Psychometric analysis

The content validity (qualitative method, CVI), construct validity (EFA, CFA), and reliability (Cronbach's alpha, test–retest method) were assessed to perform the psychometric analysis. The CVI of all items was acceptable. Item 19 was removed in EFA. The items had a good factor validity. To measure the reliability of the inventory, two approaches of test–retest and internal consistency were examined, based on both of which the reliability was reported to be good.

The CBI has been translated and psychometrically analyzed in different countries. In a methodological study conducted in Turkey, Gul and Dinc (2020) translated and psychometrically analyzed the CBI-24 with the participation of 356 nurses and 363 patients. They assessed and confirmed the validity of the inventory by evaluating the face, content, and construct validity. To assess the reliability, they utilized the internal consistency and test–retest reliability methods and they reported the Cronbach's alpha of the inventory to be 0.97 for nurses and 0.99 for patients [1].

Fenizia et al. (2019) examined the psychometric properties of the CBI-24 from the perspectives of 300 undergraduate nursing students in Italy. In their study, the EFA was conducted using Mplus maximum likelihood with GEOMIN diagonal rotation. Based on the results of their study, four dimensions of "being with", "doing with competence", "responding to individual needs", and "providing effective care" were identified for this tool. They indicated an appropriate model fit for the CBI-24 and also showed high reliability of the factors as well as a positive and significant correlation between them [5].

He et al. (2013) applied the opinions of 10 nursing professors to evaluate the semantic equivalence and content validity of the Chinese version of CBI-24. They calculated the CVI of this inventory to be 0.98. They also assessed the reliability using the internal consistency method, based on which the Cronbach's alpha coefficient of the whole inventory was found to be 0.98 for the patients and 0.96 for nurses [35].

Ghafouri et al. (2021) translated, cross-culturally adapted, and psychometrically analyzed the CBI-16 in Iran. For the psychometric analysis, they assessed the content (quantitative and qualitative) and construct validity with the participation of 509 patients. Regarding the alpha value of 0.89, the internal consistency of the inventory was shown to be good. The construct validity was assessed using the CFA and EFA. Based on the results of the EFA, the CBI-16 was loaded on two factors. The extracted factors were named "communicating respectfully" and "professional knowledge and skill". These two factors explained 50.17% of the total variance. Moreover, the CFA showed an acceptable model fit for the two factors of the CBI-16 [19].

A review of the studies conducted in this area revealed that different versions of the CBI have had good validity and reliability, which contributes to the validity of this tool. In line with the results of our study, the CVI of the CBI was measured to be higher than 0.8 in several studies and its Cronbach’s alpha was calculated to be above 0.9, all of which showed high reliability and validity of this inventory. The removal of item 19 was the only difference between the results of our study and those of other studies in this area. The researchers agreed to remove this item due to the factor loading of less than 0.3 and the reduction of Cronbach's alpha to 0.32 with the presence of this item. Item 19 is related to the satisfaction of the expressed and non-expressed needs of the patient. Cultural differences between countries may have been the reason for this discrepancy, which means that the expression of a need or pain is an embarrassing issue in the Iranian context so that patients refrain from expressing their needs and do not consider it an important issue in their treatment process.

The Persian version of the CBI-24 is a valid and reliable instrument to measure caring behaviors in both patients and nurses. The evaluation of caring behaviors in patients leads health officials to determine patient satisfaction, while the evaluation of care behaviors in nurses enables them to self-assess caring behaviors and improve the quality of care.

## Conclusion

The Persian version of the CBI-24 has acceptable validity and reliability, so it can be utilized to examine caring behaviors among Persian-speaking nurses and patients. The self- assessment of caring behaviors by nurses and patients provides the opportunity for evaluating and reflecting on the quality of caring practice and ultimately patient satisfaction. The self-assessment of caring behaviors among nurses enables health officials to monitor and improve the quality of care and this self-assessment among patients provides an opportunity to ensure patient satisfaction.

### Study strengths and limitations

The Persian version of the CBI-24 is a short and reliable instrument that imposes less burden on respondents. This inventory enables us to measure the quality of care in descriptive studies and evaluate the effectiveness of interventions in nursing research. It can be also applied in many clinical settings. Regarding the psychometric analysis of the Persian version of the CBI-24 among both nurses and patients, it allows us to compare the perceptions in both groups.

The CBI-24 can be also used in evaluating the performance of nursing students and sensitizing them to indicators of caring behaviors. Furthermore, the Persian version of CBI-24 can be used for conducting studies on nurses' performance and patient satisfaction.

The translation of the CBI-24 into Persian was conducted using a combination of the WHO model [25] and the Wild et al. (2005) approach [26] to ensure the conceptual similarity of its Persian version with the original one. In the psychometric analysis process, in addition to CVI, a modified Cohen's kappa coefficient was also calculated to strongly confirm the validity of the inventory. Moreover, a standard sample size was applied to perform factor analysis.

One of the important limitations of this study was the use of common samples for both CFA and EFA. This inventory is recommended to be psychometrically assessed in other target groups such as students and patients with psychiatric disorders.

## Data Availability

The datasets used and/or analyzed during the current study are available to be received from the corresponding author on reasonable request.

## Abbreviations

CBI: Caring Behaviors Inventory
CVI: Content Validity Index
EFA: Exploratory Factor Analysis
CFA: Confirmatory Factor Analysis
KMO: Kaiser − Meyer − Olkin
GFIs: Goodness of Fit Indices
RMSEA: Root Mean Square Error of Approximation
MSE: Mean Squared Error
CMIN/DF: Minimum value of chi-square/Degree of freedom" ratio
CFI: Comparative Fit Index
NFI: Normed Fit Index
RFI: Relative Fit Index
IFI: Incremental Fit Index
AGFI: Adjusted Goodness-of-Fit Index
PSS: Patient Satisfaction Scale
CBA: Caring Behaviors Assessment Tool
CBM: Caring Behavior Measurement
